# Determinants of interchain coupling properties of Te atomic chains

**DOI:** 10.1038/s41598-022-06750-2

**Published:** 2022-02-22

**Authors:** Jie Han, Quan Ming Li, Wang Gao

**Affiliations:** Key Laboratory of Automobile Materials, Department of Materials Science and Engineering, Ministry of Education, Jilin University, Changchun, 130022 China

**Keywords:** Electronic structure of atoms and molecules, Electronic and spintronic devices

## Abstract

The coupling effect of one-dimensional (1D) materials is of great significance for the practical application of 1D materials in high-density memory devices and ultra-micro nanometer array lasers. However, the determinants of the coupling effect remain debated. Here, using first principles methods, we investigate the effects of chirality, size and stacking mode on the stability and electronic properties of few-chain Te nanowires. We find that the stacking mode and size play a dominant role in the stability of the nanowires, while the chirality and size have a key effect on the electronic structures. These phenomena are mainly due to the quantum size effect and the special helical structure of the Te chain. Our findings provide the means for adjusting the band gap and the candidates for constructing n-type spin devices, which serve as a basis for the research and manufacture of new nano electronic devices.

## Introduction

1D materials have recently attracted great attention because of their unusual properties caused by the reduction of dimension. On the one hand, 1D nanomaterials are an ideal system to study the relationship between electronic transport properties, optical, magnetic and size in basic research^1–9^. On the other hand, the specific geometry of 1D nanomaterials plays an important role in the construction of nano electronic and optical devices, which can be used as the minimum dimension structure for effective transmission of electrons, lasers and field effect transistors in the practical applications^10–12^. Therefore, it is crucial to understand the coupling effect of 1D materials for the practical applications of new electronic devices^13–17^. In fact, the coupling of low-dimensional materials has an effect on the electronic structure such as: in 2D materials^18,19^. For instance, in the 2D limit of three-dimensional (3D) topological insulators, a finite energy gap has been discovered because of the coupling between the surface states^20^. Owing to the coupling between monolayer graphene and few-layer semiconducting tungsten disulphide, it is observed that the spin orbit coupling (SOC) of graphene acquires 17 meV^21^ without modifying the structure of graphene, which is three orders of magnitude higher than its intrinsic value. Although studying the coupling characteristics of 2D materials is helpful to understand the coupling between 1D materials, few studies have been done on the coupling between nanowires.

Low-dimensional materials, especially 1D systems, have poor stability due to their large specific surface. Therefore, it is very difficult to synthesize and study 1D few-chains in experiments. Different from the common 3D materials with covalent network structures, Te bulk is composed of a 1D helical chiral Te chain through non-covalent bonds, in which the helical chains are parallel to the c-axis and interact with each other only by weak van der Waals force^22–25^. The structure of Te composite makes it easier to obtain 1D nanostructure than other elements. In the density functional theory (DFT) study of the atomic Te single chain, we found that 1D helical Te single chain exhibits a good dynamical and thermodynamic stability^26^. The 1D helical Te chain has 1D semiconducting bands with a giant Rashba splitting, and the Rashba parameters can be adjusted by the strain. The previous theoretical studies have found that the chirality and phase sequence of the 1D telluryne can regulate the carrier transport type and direction^27^. However, 1D Te nanowire is difficult to exist as a single chain in reality, and in most cases, it forms multiple chains. Recently, some research groups have successfully synthesized few-chain Te nanowires by the confinement effect of nanotubes^28,29^, whose electronic properties strongly depend on the diameter of nanotubes. The current density of Te nanowires encapsulated in boron nitride nanotubes (BNNT) is 1.5 × 10^8^ A/cm^2^, which is higher than that of most semiconductor nanowires. In addition, Te nanowires encapsulated in BNNTs can be used to create field-effect transistors with a diameter of only 2 nm, which is suitable for spin field effect transistors. From the perspective of practical application, it is necessary to understand the interchain coupling properties of Te atomic chains. Considering the promising properties of 1D Te chain and the maturity of its preparation, it is very significant and meaningful to explore the coupling between nanowires chains by one typical 1D prototype—1D few-chain Te nanowires.

In this contribution, we have studied the effects of the chirality, size and stacking mode on the stability and electronic properties of the few-chain Te nanowires. The phonon spectra and interchain binding energy suggest that helical Te chains are both dynamically and thermodynamically stable. We find that the bulk stacking mode and larger size always lead to higher stability, regardless of the chirality. In addition, the band gap and Rashba parameters decrease with the increase of the number of chains. Due to the nature of the heavy elements of Te, the spin–orbit coupling effect is considered when studying the electronic structure. Furthermore, changing the chirality and the stacking mode can adjust the value of band gap and Rashba parameters of the 1D helical Te chains. These phenomena are mainly due to the quantum size effect and the special helical structure of the Te atomic chains. Our findings provide a simple way to adjust the electronic structure of the Te atomic chains, which can serve as the basis for the research and manufacture of new nano electronic devices.

## Results and discussion

### Energetic stability and dynamical stability of helical Te chains

Our previous work has shown that 1D helical Te chains exhibited giant Rashba splitting systems with strict 1D characters^26^. Based on a single 1D helical Te chain, we study the electronic structure with the superposition of multiple chains. To better understand the influence of multiple chain coupling, we study the effects of chirality, size and stacking modes on the electronic properties of the few-chain Te nanowires.

**Figure 1:**
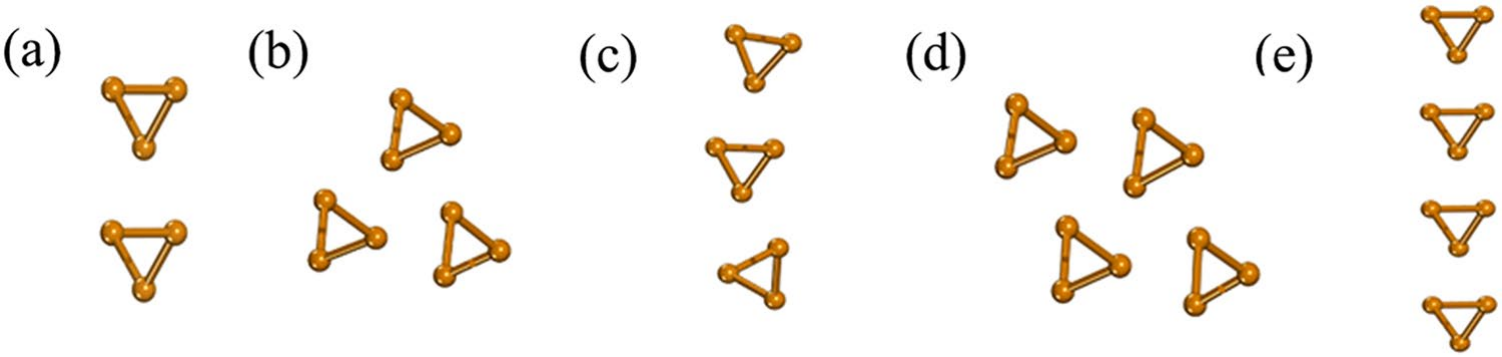
Atomic structures of the same chirality Te chains (**a**) S-2-1, (**b**) the bulk stacking mode S-3-1, (**c**) the linear stacking mode S-3-2, (**d**) the bulk stacking mode S-4-1 and (**e**) the linear stacking mode S-4-2. Use office2013 version to create images and the link of the software is: https://zbhrj1.jlu.edu.cn/download/office2013.html.

**Figure 2:**
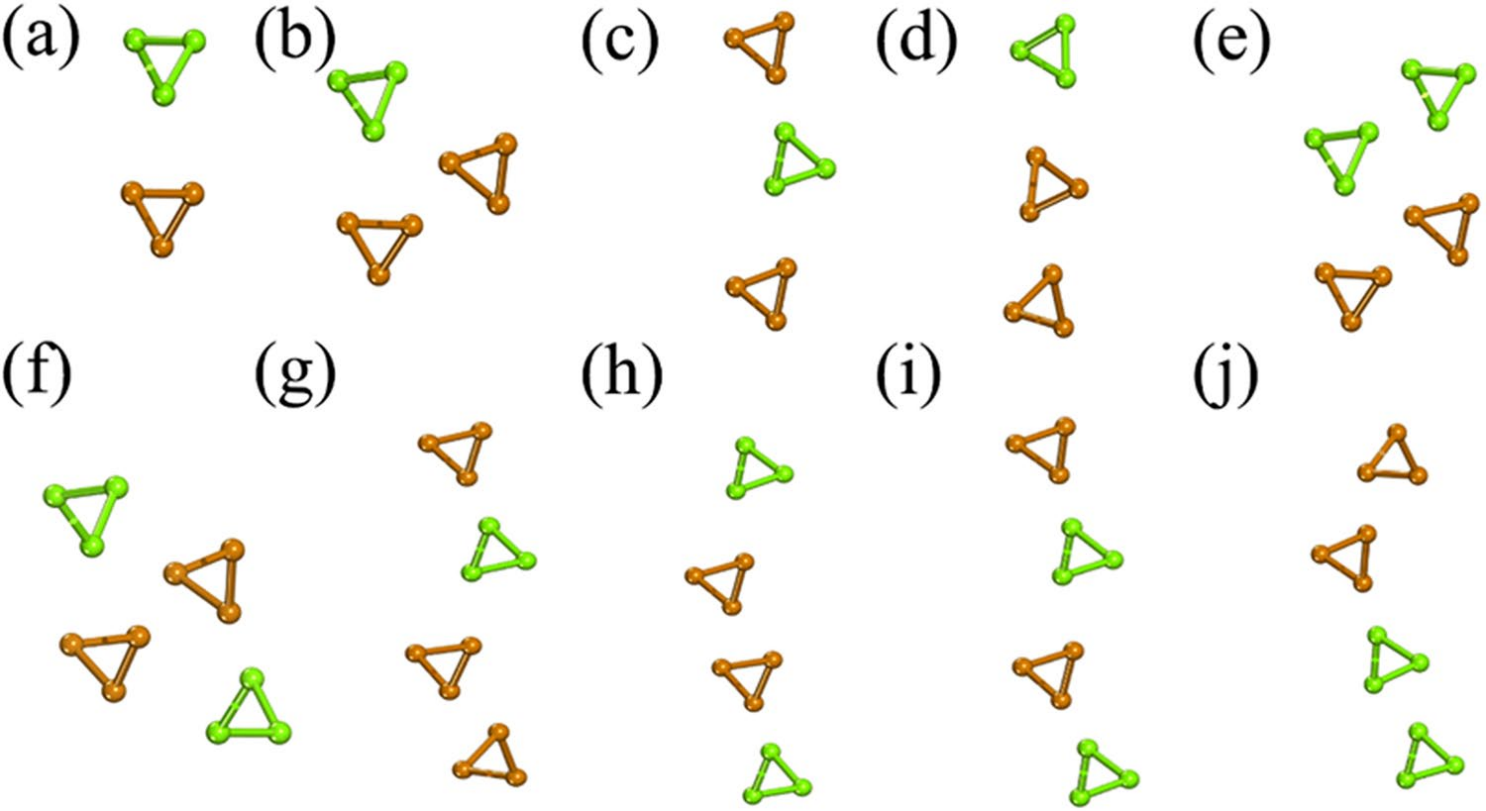
Atomic structures of different chirality Te chains: (**a**) D-2-1, (**b**) the bulk stacking mode D-3-1, (**c**) the linear stacking mode D-3-2, (**d**) the linear stacking mode D-3-3, (**e**) the bulk stacking mode D-4-1, (**f**) the bulk stacking mode D-4-2, (**g**) the linear stacking mode D-4-3, (**h**) the linear stacking mode D-4-4, (**i**) the linear stacking mode D-4-5 and (**j**) the linear stacking mode D-4-6 the few-chain Te nanowires composed of single chains with different chirality, green to represent one chirality feature, mustard yellow to represent the other. Use office2013 version to create images and the link of the software is: https://zbhrj1.jlu.edu.cn/download/office2013.html.

**Figure 3:**
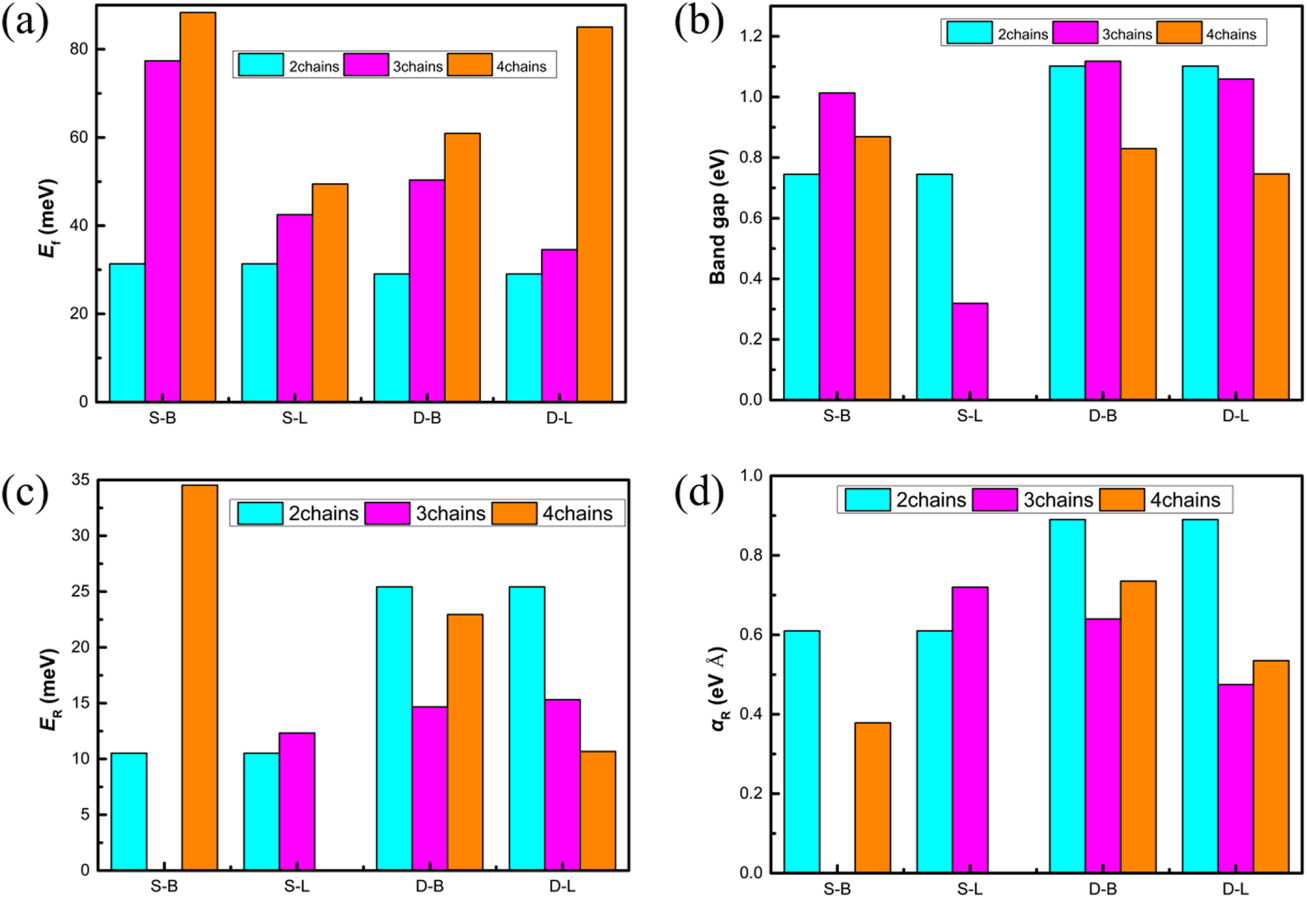
Variations of the formation energy *E*_f_ in meV per atom (**a**), band gap in eV (by PBE with SOC) (**b**), Rashba energy E_R_ in meV (**c**), and Rashba parameter α_R_ in eV Å (**d**) with respect to the size for the Te chains. Use Adobe Photoshop to create images and the link of the software is: https://zbhrj1.jlu.edu.cn/download/Adobe_Photoshop.html.

As known, the stability of materials is the basis of further application. Hence, before analyzing electronic structure, we consider the stability of the few-chain Te nanowires. From the perspective of chirality, we consider the same chirality (S-chirality) and different chirality (D-chirality). In the case of the size effect, we increase the number of the Te chains from two to four (named: -2-, -3- and -4-). For the stacking modes, we consider the linear stacking mode (L-stacking mode) and the bulk stacking mode (B-stacking mode). The structures of few-chain Te nanowires composed of 2–4 Te single chains with the same chirality are named S-2-1, S-3-1, S-3–2, S-4-1 and S-4-2, respectively (see Fig. 1). Among the few-chain Te nanowires composed of single chains with different chirality, we use green to represent one chirality feature, mustard yellow to represent the other, and name them D-2-1, D-3-1, D-3-2, D-3-3, D-4-1, D-4-2, D-4-3, D-4-4, D-4-5 and D-4-6 (as shown in Fig. 2). When the chirality, stacking mode and size are the same, we also consider the different phase sequences that correspond to the different relative positions between the neighboring helical chains (see Fig. S1). We calculate six phases with the same chirality, stacking mode and size, and select the phase with the lowest formation energy for the analysis of electronic properties. We adopt the formation energy to estimate the stability of the few-chain Te nanowires as:

1 $$E_{{\text{f}}}= \frac{{xE_{{{\text{single}}}} \, - E_{total} }}{3x}$$

where *E*_single_ is the total energy of a single helical Te chain, *E*_total_ is the total energy of the few-chain Te nanowires, and x is the number of the Te chains. As the size increases, the formation energy increases in Fig. 3(a). This result also indicates that the stability of few-chain Te nanowires increases with the increase of size from Fig. 3(a). For the S-chirality, the formation energies of the L-stacking mode Te nanowires are 31–49 meV/atom, which is lower than those of the B-stacking mode (77–88 meV/atom). For the D-chirality, both formation energies decrease, but the relative stability remains unchanged. However, the few-chain Te nanowires of both stacking modes are stable. In addition, we test the dynamical stability of each few-chain Te nanowires by analyzing the phonon calculations in Figs. S2–S4 of Supplemental Materials (SM). Clearly, there is no virtual frequency in the phonon spectra of all the structures, which confirms the dynamical stability of the few-chain Te nanowires.

Moreover, we study the relationship between stability and geometric structure. To obtain a thorough knowledge of the stability of few-chain Te nanowires, we then studied the effects of structural changes such as bond length and bond angle on the stability of the material. Figure S6 shows that with the increase of the number of chains, the bond length increases and the bond angle decreases. In general, when the bond length of Te nanowires is close to the bond length of the free Te chain, the bond energy will be higher and the system is more stable. The bond length and the bond angle of the free single Te chain are 2.76 Å and 103.0°^26^. When the chains are coupled, the bond length increases from 2.77Å  to 2.83 Å and the bond angle decreases from 100.6° to 99.1° with the increase of the size. Although the bond length increases and the bond angle decreases with the increase of the size, the stability of the chains increases (see Fig. 3(a)). In addition, we find that the bond lengths and bond angles are all nonuniform values in the calculated structure, while this non-uniformity increases with the increase of size. The inhomogeneity of bond length and bond angle will lead to internal strain, affect the electronic structure of the material. When the strain parameter is larger than a critical value, new phases may appear^30^.

### Electronic properties of helical Te chains

To explore the potential applications of few-chain Te nanowires in electronics and spintronics, we now turn to study the effects of chirality, size and superposition mode on the electronic properties of few-chain Te nanowires. The calculated formation energies, band gap and the Rashba parameters are shown in Fig. 3.

Clearly, the indirect band gap feature is retained regardless of the stacking modes, size or chirality, and both the conduction band minimum (CBM) and valence band maximum (VBM) are located at the G-Z for all considered Te nanowires except S-4-2(the S-chirality L-stacking mode). It is easily seen that the band gap decreases from 1.01 eV of 3 chains to 0.87 eV of 4 chains for the S chirality B-stacking mode, and it decreases from 1.06 eV of 3 chains to 0.75 eV of 4 chains for the D chirality B-stacking mode (Fig. 3(b)). This result indicates that the increase of the size of the B-stacking mode results in the decrease of band gap. Furthermore, in Fig. 3(b) the band gap also decreases in the L-stacking modes with the increase of size due to the splitting of the valence band and the conduction band induced by the chains coupling. With the increase of the size, the band gap of few-chain Te nanowires decreases from 0.32 eV to zero for the S chirality L-stacking mode, and it decreases from 1.12 eV to 0.745 eV for the D-chirality L-stacking mode. These findings reflect the revolution from the discrete energy levels to the energy band for nanomaterials. At the nanoscale, the number of overlap of orbits or energy levels increases and the thickness of the band becomes thicker with the increase of the size. This lead to the decrease of the band gap. Clearly, size can be used as a means to adjust the band gap in practical application.

Furthermore, the few-chain Te nanowires with the D chirality always generate a larger band gap than that with the S-chirality (see Fig. 3(b)). Previous advances have suggested that the electronical properties of quasi 1D structures such as single-walled carbon nanotubes^31^ depend on their diameter and chirality. Similarly, we find that the band gap can be controlled by changing the chirality and size of the few-chain Te nanowires. The semiconductor properties of spin materials play a decisive role in the fabrication of spin electronic devices. In particular, due to the special chiral structure of Te chains, the band gap can be opened by adding a helical Te chain with the opposite chirality to the previous few-chain Te nanowires in the S-chirality L-stacking mode. Such as the D-chirality few-chain Te nanowire (D-4-1 to 6) can be obtained by adding an opposite chiral helix Te chain to the few-chain Te nanowire (S-3-1). Compared with the method of changing the chirality, it is more convenient to control the band gap by peeling off a single chain Te nanowire to diminish the size of the system.

**Figure 4:**
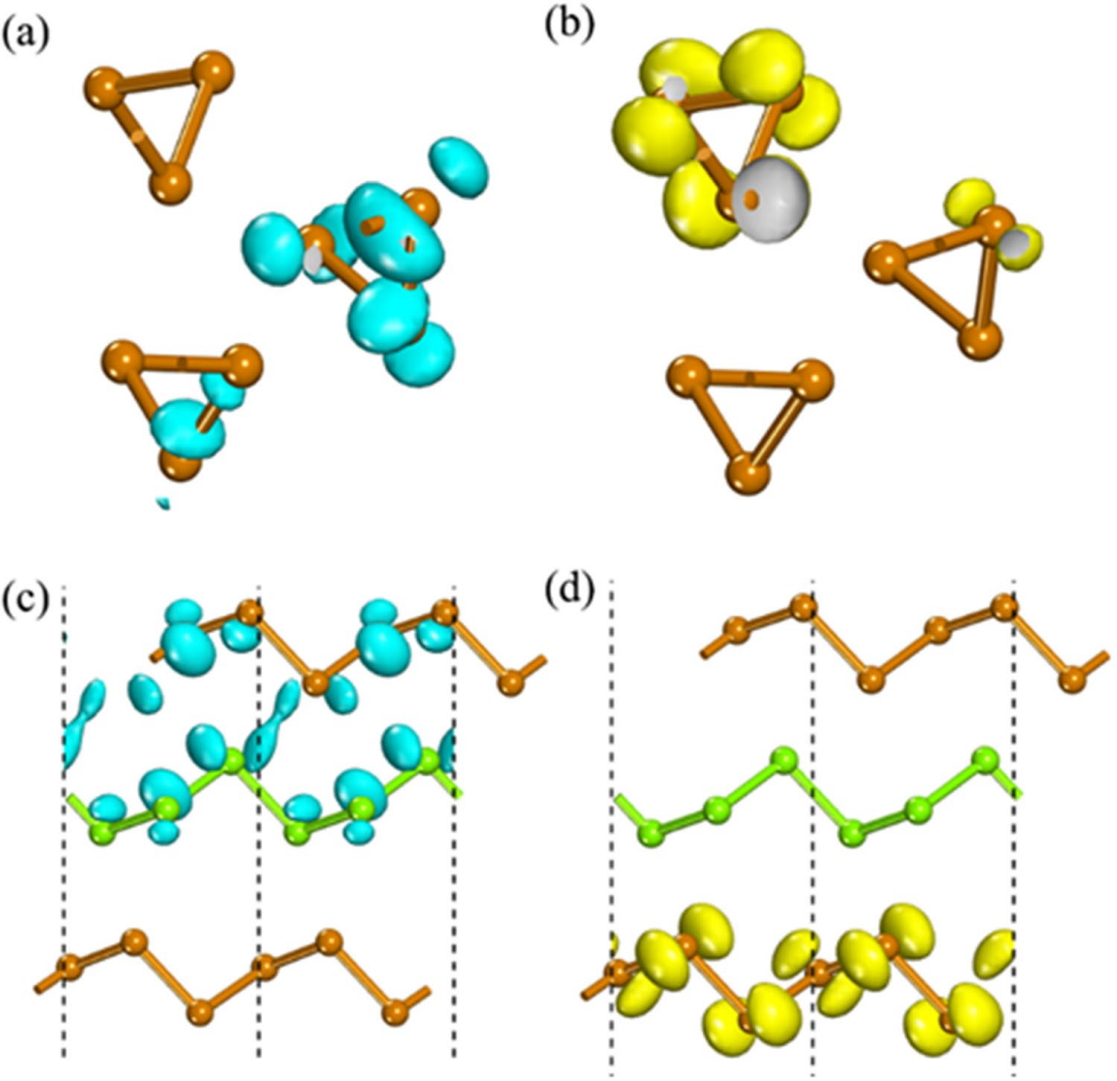
The charge density corresponding to the CBM (**a**) and VBM (**b**) of the D-3-1 and the CBM (**c**) and VBM (**d**) of the D-3–3, respectively. The isovalue is 0.004 e Bohr^−3^. Use office2013 version to create images and the link of the software is: https://zbhrj1.jlu.edu.cn/download/office2013.html.

In Fig. 3(b), the few-chain Te nanowires with the B-stacking mode always generate larger band gap than the L-stacking mode. This may be due to the periodicity of bulk Te in three dimensions, but the nanowires have periodicity only in one dimension, and the interaction strength between Te nanowires decreases for the outmost Te nanowires due to the reduction of neighboring Te nanowires. In Fig. 4, it shows the isosurfaces of the charge density corresponding to the CBM and VBM of different chirality Te nanowires in the B-stacking modes and the L-stacking modes. In the B-stacking mode, there is no overlap in CBM and VBM, indicating negligible charge transfer. In contrast, there is a significant overlap of CBM in the L-stacking mode, which implies a significant charge transfer. Clearly, the detailed spatial matching between the different chains may determine the orbital hybridization, thus the stacking modes have a significant influence on the band structures, especially the band gap.

**Figure 5:**
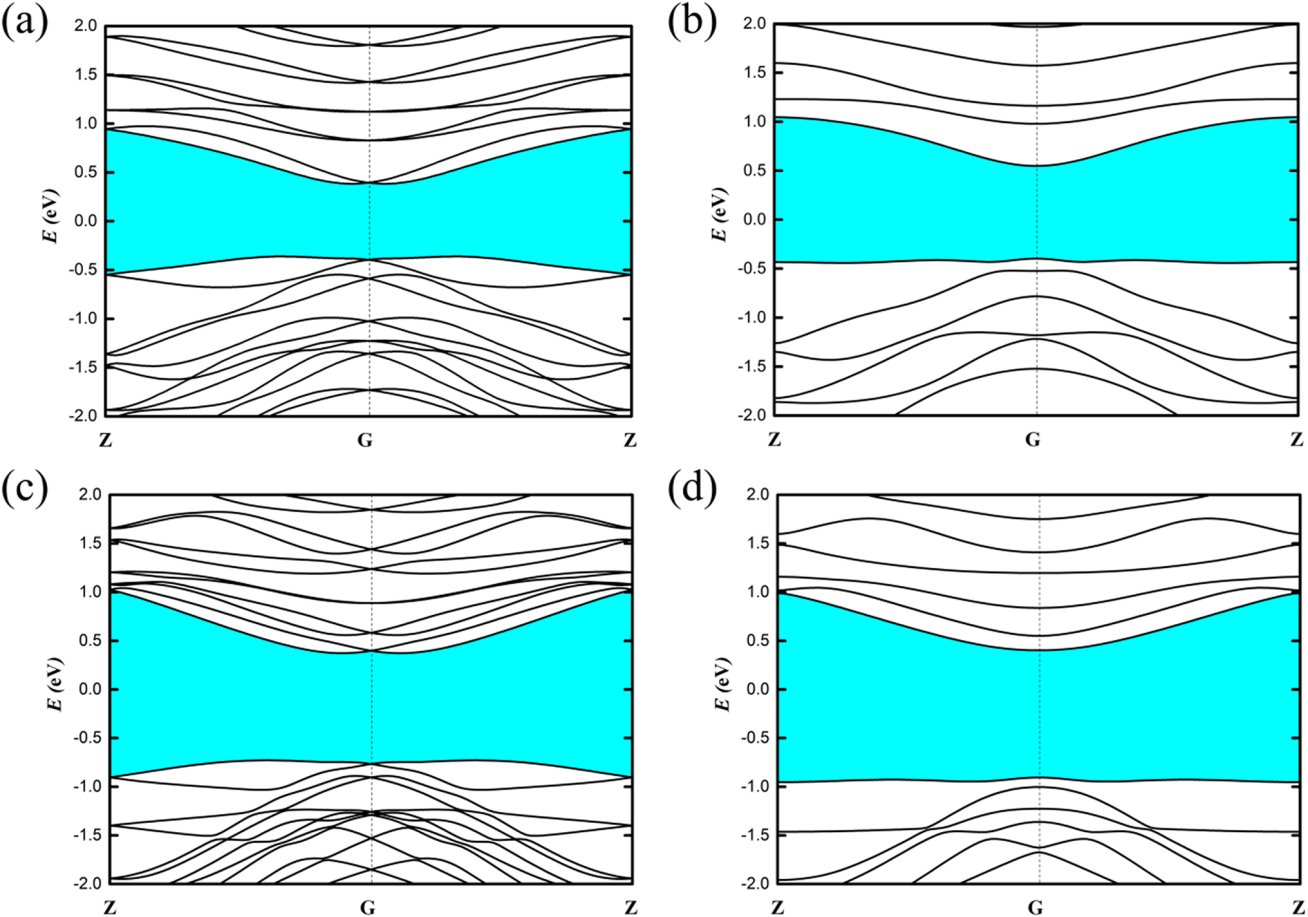
The band structure of S-2-1 with SOC (**a**) and without SOC (**b**). The band structure of D-2-1 with SOC (**c**) and without SOC (**d**). Use Adobe Photoshop to create images and the link of the software is: https://zbhrj1.jlu.edu.cn/download/Adobe_Photoshop.html.

The effect of SOC is investigated because of the nature of the heavy element Te. Figure 5(a), (b) and (c), (d) show the band structure of PBE with/without SOC. A strong spin splitting is clearly observed in the band structures of the S-2-1 and the D-2-1 with the inclusion of SOC (see Fig. 5(a), (c)). Clearly, the effect of SOC reduces the band gap by about 0.2 eV. More interestingly, the SOC effect in the few-chain Te nanowires not only affects the band gap, but also induces a giant band splitting, with the CBM and the VBM locating off the high-symmetry point and forming a Rashba-type band splitting, which may be applied into spintronics. We calculate three key Rashba splitting parameters (*E*_R_, Δ*k*_R_ and *α*_R_) to quantitatively study the strength of Rashba spin splitting for 1D helical chains. The Rashba energy (*E*_R_) is the energy difference between the band edge and the crossing point, the Rashba momentum offset Δ*k*_R_ is the moment splitting of the Rashba bands from the crossing point, while the Rashba constant *α*_R_ represents the strength of the Rashba effect and is calculated by using the equation *α*_R_ = 2*E*_R_ /Δ*k*_R_. Materials with large Rashba constant and large Rashba energy offer us more opportunities to adjust their spintronic properties. Herein, our calculations show that the Rashba parameters of CBM are *E*_R_ = 10.51 meV, *K*_R_ = 0.03 Å^-1^, and *α*_R_ = 0.61 eV Å for S-2–1, while those are *E*_R_ = 25.42 meV, *K*_R_ = 0.06 Å^-1^, and *α*_R_ = 0.89 eV Å for D-2–1.

We first study the widely discussed Rashba constant. The D-chirality structures lead to bigger Rashba constants than the S-chirality ones in the B-stacking mode, as shown in Fig. 3 and Table S1. The characteristics of the D-chirality in the B-stacking modes will aggravate an asymmetry of the system, and the symmetry of the structure is closely related to the Rashba constants. Therefore, it is easy to understand that the Rashba constants of the D-chirality are larger than those of the S-chirality in the B-stacking mode. However, there is a different rule in the L-stacking mode. In L-stacking mode, the S-chirality structures lead to bigger Rashba constants than the D-chirality ones. The stacking modes and chirality will have a coupling effect on the Rashba constants of the Te nanowires.

Obviously, the Rashba splitting in the B-stacking mode appear at both the CBM band and the VBM band, but in the L-stacking mode only split at the CBM band, which makes only electrons experience the Rashba splitting in the L-stacking mode. Therefore, this property can be used to develop advanced functional spintronic devices (n-type electronic devices), where the L-stacking modes Te nanowires can be used for blocking the undesired p-type spins. These findings reflect the possibility of adjusting the Rashba effect of the Te nanowires by the stacking mode.

Compared with the conventional Rashba systems, the few-chain Te nanowires are conducive to adjust the band gap and the carrier type. In addition, the *E*_R_ of D-2-1 reaches 25.42 meV, which is three or four times larger than the other Rashba systems such as InGaAs/InAlAs (< 1 meV)^32^, and Si(557)-Au nanowire (N/A)^33^. Furthermore, the *E*_R_ (*α*_R_) of few-chain Te nanowires is comparable with that of the single isolated helical Te chain 24 meV (0.84 eV Å)^26^. However, the *E*_R_ (*α*_R_) of few-chain Te nanowires are smaller than that of 1D nanowires reported in some literatures, such as the *E*_R_ (*α*_R_) in the Pt/Si(111) nanowire is 81 meV (1.36 eV Å)^34^, Q2 structure for Bi on In/Si(111) is 78.2 meV (2.1 eV Å), and H1 structure for Bi on In/Si(111) is 59.6 meV (3.1 eV Å)^35^. Due to the existence of surface dangling bonds or the presence of the (semi) metallic surface states, the performance of these semiconductor nanowires degraded. Thus, these nanowires are disadvantageous in spintronic devices. On the contrary, the few-chain Te nanowires can maintain the semiconductor state without dangling bonds and are crucial to the development of spintronic devices.

## Discussion

Our results show that the chirality, stacking mode and size have significant effects on the stability and electronic structures of the 1D few-chain Te nanowires. The few-chain Te nanowires are both dynamically and thermodynamically stable. Regardless of the chirality, the B-stacking mode and larger size always lead to higher stability. In addition, the chains with the S-chirality are more stable than those with the D-chirality. On the other hand, regardless of the chiral mode, the band gap of the B-stacking mode is larger than that of the L-stacking mode. For the nanowires with the L-stacking mode, the D chirality can enlarge the band gap and Rashba parameters relative to the S-chirality. In addition, the band gap and Rashba parameters increase with the decrease of size. As far as we know, the studied Te chains have some characteristics which are obviously different from the previously suggested 1D structure. First of all, the helical Te atomic chain that forms the 1D few-chain Te nanowires is an atomic 1D structure without a dangling bond. Secondly, by adjusting the chirality and the stacking mode, one can make the 1D few-chain Te nanowires undergo a transition from metal to semiconductor and obtain the linear ones with the larger band gaps and Rashba parameters. Moreover, we are able to obtain the n-type spinors in few-chain Te nanowires with the L-stacking mode that only electrons experience the Rashba splitting. Compared with the well-known 1D chiral systems, these Te chains exhibit broad potential application in spintronic devices.

Lastly, we discuss the possibility of obtaining 1D few-chain Te nanowires experimentally. In recent years, thin Te nanosheets and nanowires have been successfully prepared by van der Waals epitaxy^25^, solution phase growth^36^, and vapor phase reactions^37^. Although the ultra-thin Te chains have been exfoliated in experiments^38^, the diameter of the obtained thinnest Te chains is about 1–2 nm, which is the combination of a large number of helical Te atomic chains, rather than the chains we propose. It is still present a challenge to control the thickness uniformity of exfoliated products and remove the small-scale derived materials^25^, especially it is difficult to generate 1D Te chains with specific stacking modes or chiral structures. Therefore, in addition to the exfoliating method, we expect other better methods to synthesize the Te chains. A potentially effective way to synthesize the 1D Te chains is taking advantage of the confinement effect of nanotubes, which can regulate the structure of Te chains depending on the diameter of nanotubes. In experiments, the helical Te atomic chain has been successfully obtained by single-walled carbon nanotube (SWCNT) encapsulation^28^. Single atomic chain and the few-chain limited Te nanowires have been synthesized by a physical vapor transport (PVT) technique by filling the cavities of CNTs and BNNTs^29^, respectively. The electrically insulated BNNTs could provide an ideal protective layer for the Te chain in the cavity without affecting the electronic structure of the inner layer^26,29^. The B-stacking mode of few-chain Te nanowires has been successfully produced in experiments by using BNNTs encapsulation, while the L-stacking mode one could be synthesized between 2D structural interlayers.

## Conclusion

In summary, we have studied the effect of chirality, size and stacking mode on the stability and electronic structures of few-chain Te nanowires using DFT methods. Our results demonstrate that the few-chain Te nanowires are both dynamically and thermodynamically stable and the B-stacking mode and larger size always lead to higher stability, regardless of the chirality. Furthermore, the band gap of the few-chain Te nanowires decreases with the increase of the size, whereas the band gap of the B-stacking mode is larger than that of the L-stacking mode. In addition, the D-chirality structures lead to bigger Rashba parameters than the S-chirality in the B-s modes, and only n-type Rashba splitting exists in the linear stacking mode. Our findings provide the means for adjusting the band gap and the candidates for constructing n-type spin devices, which serve as a basis for the development of new nano electronic devices. Despite these achievements, there are still many challenges in the synthesis of the few-chain Te nanowires. To accurately synthesize the chiral nanowires, we look forward to appropriate structural templates or new experimental methods.

## Methods

All calculations were performed by using the all-electron FHI-aims code with a “tight” basis set^39^, except the phonon spectra that were done with CASTEP code^40^. We obtained the geometric and energetic details using Perdew-Burke-Ernzerhof^41^ (PBE) augmented with the Tkatchenko-Scheffler (PBE + TS)^42^. To eliminate the interaction between the periodic nanowire structures, we build structures with a vacuum of 100 Å to separate the periodic images of the nanowires by aims code. The test convergence shows that the k-point grid of 1 × 1 × 2 is accurate sufficiently for the calculations. The k-grid Z to G (0.000 0.000 0.500, 0.000 0.000 0.000) is used to calculate the band structure for the 1D nanowires, which includes 48 points between these two points, thus the band structure tends to be smooth and reliable as much as possible. The SOC effect was included for the calculations of the more accurate values of band gaps. All of these nanowires are composed of isolated helical Te chain, and the nanowires are periodic only in the c-axis direction. For the phonon calculations with CASTEP, the extensive tests allowed us to adopt the energy cutoff of 600 eV for the plane wave basis set and the 2 × 2 × 10 Monkhorst–Pack K-points for sampling the first brillouin zone because phonon calculations need more intensive k-point. Overall, the geometric configurations were optimized until the maximum force in chain of the directions was less than 0.01 eV/ Å, and the convergence of total energy was less than 1 meV/ atom.

## Supplementary Information

Supplementary Information.
