# Supplementary material for: Determinants of interchain coupling properties of Te atomic chains
Source: Sci Rep. 2022 Feb 22;12:2973. doi: 10.1038/s41598-022-06750-2 (PMC8863999; doi:10.1038/s41598-022-06750-2)
Supplement: Supplementary file 1 — Supplementary Information. [file 41598_2022_6750_MOESM1_ESM.docx]

Supplementary Materials for

**Determinants of Interchain Coupling Properties of Te Atomic Chains**Jie Han, Quan Ming Li,^a*^ and Wang Gao,^a*^

**^a^**.Key Laboratory of Automobile Materials, Ministry of Education, Department of Materials Science and Engineering, Jilin University 130022, Changchun, China.

*Corresponding author. Email: lqm@jlu.edu.cn; wgao@jlu.edu.cn.


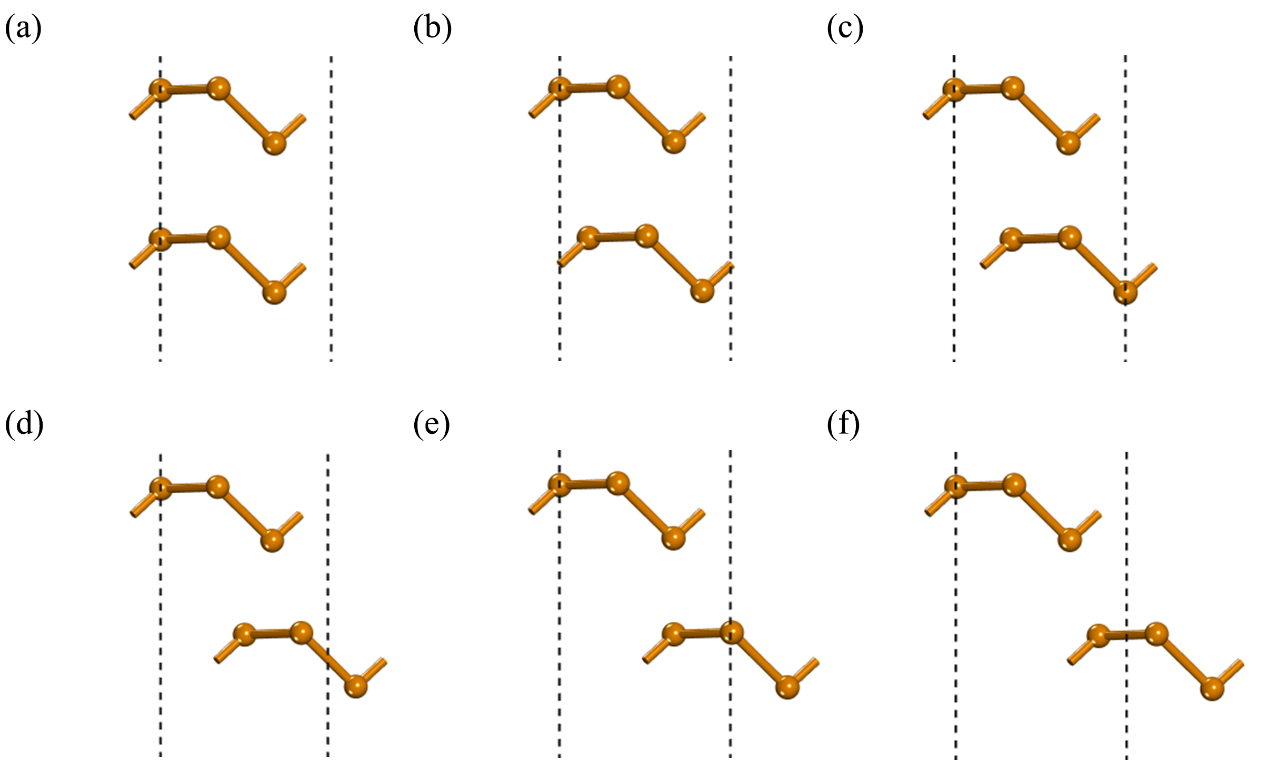


Figure S1. Atomic structures of the S-2-1 six comparable phase, each chain is shifted half a phase backward from the previous one. Use office2013 version to create images and the link of the software is: https://zbhrj1.jlu.edu.cn/download/office2013.html


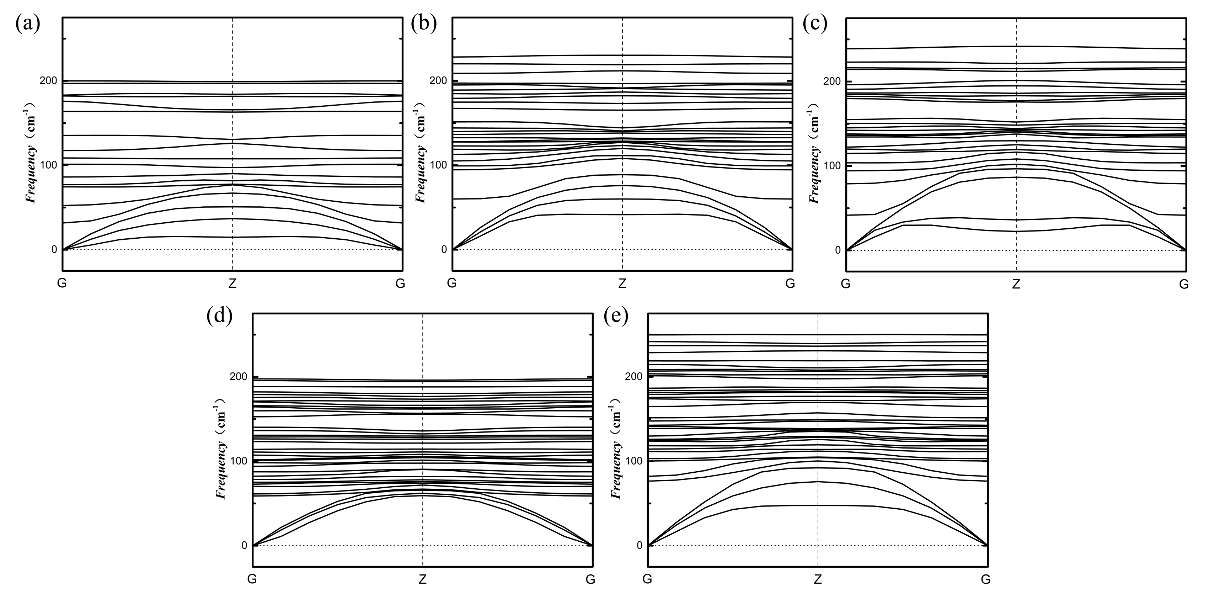


Figure S2. The phonon spectra of (a) S-2-1, (b) S-3-1, (c) S-3-2, (d) S-4-1 and (e) S-4-2. Use Adobe Photoshop to create images and the link of the software is: https://zbhrj1.jlu.edu.cn/download/Adobe_Photoshop.html.


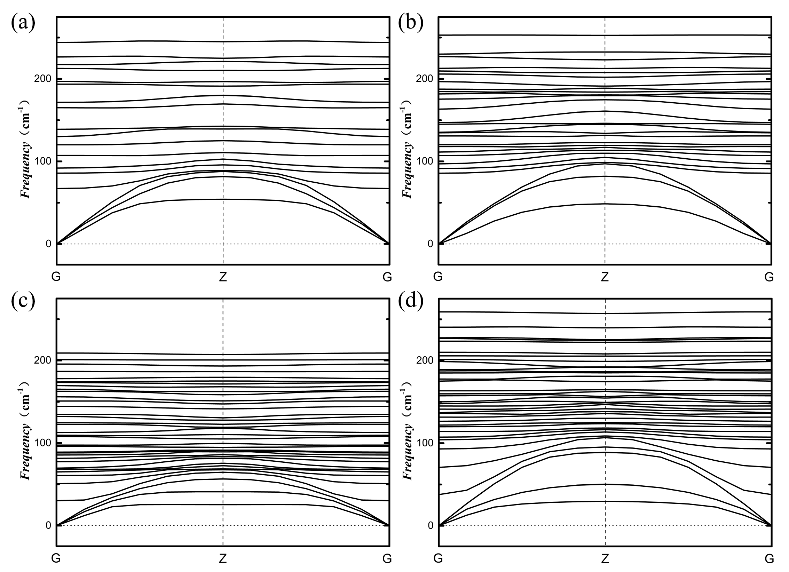


Figure S3. The phonon spectra of (a) D-2-1, (b) D-3-1, (c) D-4-1 and (d) D-4-2. Use Adobe Photoshop to create images and the link of the software is: https://zbhrj1.jlu.edu.cn/download/Adobe_Photoshop.html.


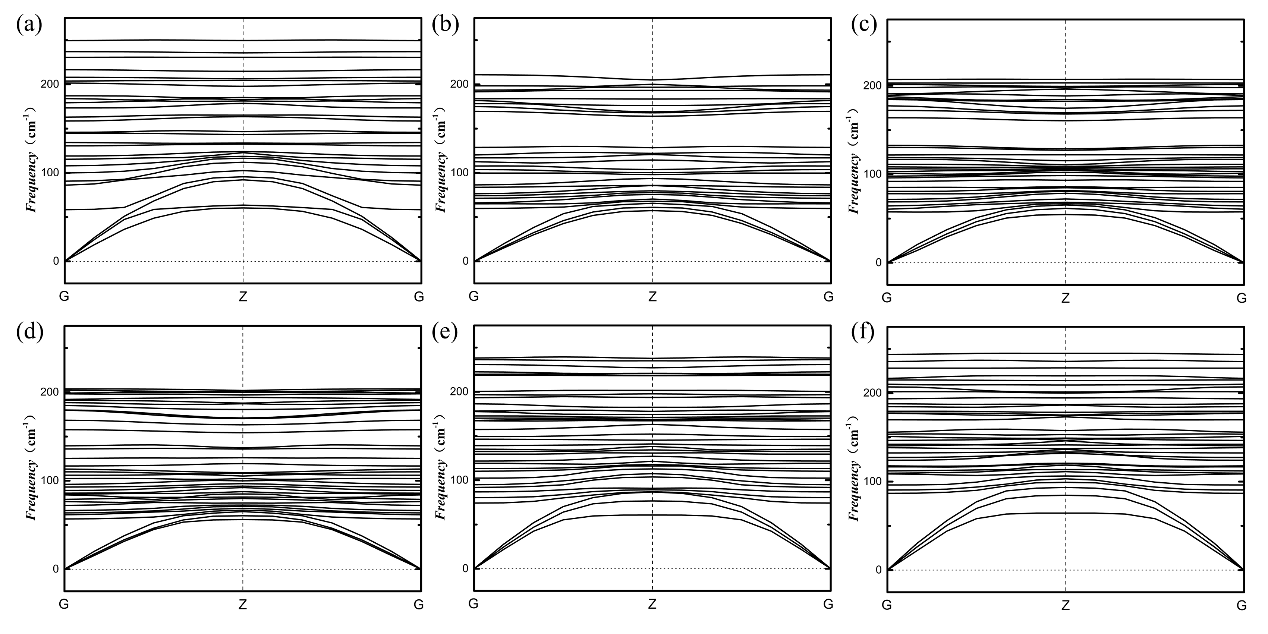


Figure S4. The phonon spectra of (a) D-3-2, (b) D-3-3, (c) D-4-3, (d) D-4-4, (e) D-4-5 and (f) D-4-6. Use Adobe Photoshop to create images and the link of the software is: <https://zbhrj1.jlu.edu.cn/download/Adobe_Photoshop.html>.

To study the dynamics stability of Te chain, we calculate phonon spectra. Our phonon results demonstrate that isolated helical Te chain is dynamically stable because of the absence of imaginary frequency phonon modes and the separation of acoustic and optical branches.

Table S1. Rashba energy E_R_ in meV, momentum offset ΔK_R_ in Å^-1^, Rashba parameter α_R_ in eV Å, band gap in eV (by PBE with SOC and PBE without SOC), and the formation energy in meV per atom for the different chirality ten different stacking Te chains.

|  | E_R_ | K_R_ | α_R_ | Band gap（PBE+SOC, PBE） | E_formation_ | |
| --- | --- | --- | --- | --- | --- | --- |
| **D-2-1** | 25.42 | 0.06 | 0.89 | 1.10, 1.31 | -29.06 |  |
| **D-3-1** | 14.67 | 0.05 | 0.64 | 1.12, 1.21 | -50.34 |  |
| **D-3-2** | 21.01 | 0.07 | 0.61 | 1.17, 1.30 | -34.10 |  |
| **D-3-3** | 9.62 | 0.06 | 0.34 | 0.95, 1.11 | -35.00 |  |
| **D-4-1** | 7.17, 9.34 | 0.03, 0.03 | 0.42, 0.55 | 0.75, 0.93 | -63.22 |  |
| **D-4-2** | 36.56 | 0.08 | 0.92 | 0.91, 1.11 | -58.63 |  |
| **D-4-3** | 11.73 | 0.05 | 0.51 | 0.99, 1.06 | -41.46 |  |
| **D-4-4** | ~ | ~ | ~ | 1.06, 1.10 | -41.02 |  |
| **D-4-5** | ~ | ~ | ~ | 0.61, 0.74 | -40.50 |  |
| **D-4-6** | 9.61 | 0.03 | 0.56 | 0.32, 0.52 | -47.05 |  |





Figure S5. Variations of the Rashba momentum offset Δ*k*_R_ with respect to the size for the Te chains. Use Adobe Photoshop to create images and the link of the software is: https://zbhrj1.jlu.edu.cn/download/Adobe_Photoshop.html.


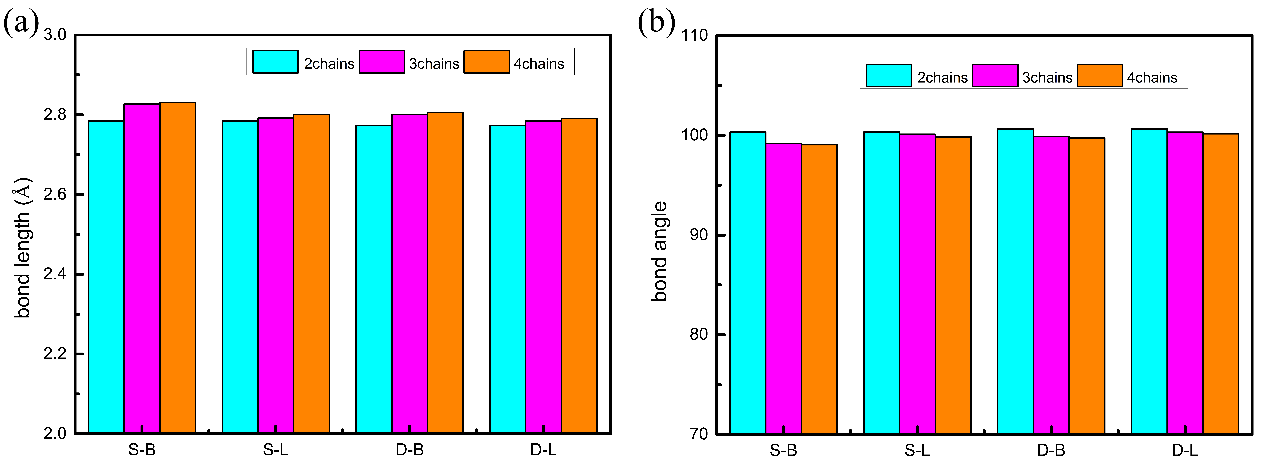


Figure S6. Variations of the bond length (a) and bond angle (b) with respect to the size for the Te chains. Use Adobe Photoshop to create images and the link of the software is: https://zbhrj1.jlu.edu.cn/download/Adobe_Photoshop.html.


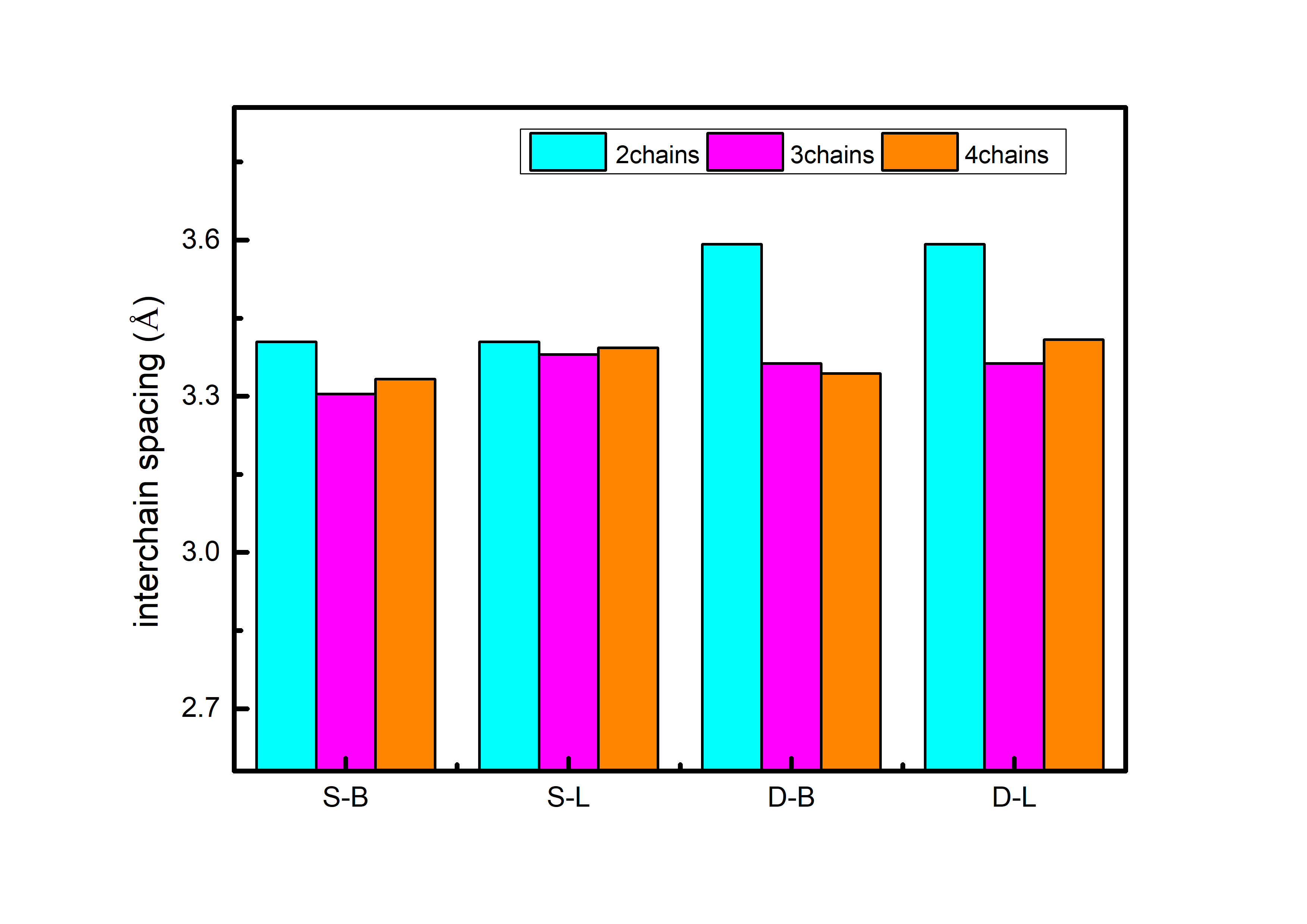


Figure S7. Variations of the interchain spacing with respect to the size for the Te chains. Use Adobe Photoshop to create images and the link of the software is: https://zbhrj1.jlu.edu.cn/download/Adobe_Photoshop.html.
